# A novel missense variant expands the phenotype and genotype of *PAX6*-associated foveal hypoplasia accompanied by various manifestations of anterior segment dysgenesis

**DOI:** 10.1186/s12886-023-03054-5

**Published:** 2023-08-08

**Authors:** Yanping Yu, Hongyan Jia, Qian Ma, Ranran Zhang, Yonghong Jiao

**Affiliations:** Beijing Tongren Eye Center, Beijing Tongren Hospital, Capital Medical University, No. 1 Dongjiaominxiang Street, Dongcheng District, Beijing, 100730 China

**Keywords:** *PAX6* gene, Foveal hypoplasia, Anterior segment dysgenesis, Symmetrical corectopia

## Abstract

**Background:**

According to previous reports, *PAX6*-associated foveal hypoplasia (FH) could usually be accompanied by various anterior segment anomalies including variable iris changes. This study aims to exhibit unusual phenotypes of a novel missense variant of *PAX6* from a Chinese pedigree.

**Methods:**

Ophthalmic examinations including slit-lamp biomicroscopy, gonioscopy, ophthalmic ultrasound, ultrasonic biomicroscopy, optical coherence tomography, wide-field fundus imaging, and visual field test were performed to evaluate the clinical manifestations. Whole-exome sequencing (WES) and bioinformatics analysis were conducted in eight members from this pedigree to identify the causative mutation.

**Results:**

WES revealed a novel heterozygous substitution of *PAX6* (NM_000280.5:c.157G > A, p.(Val53Met) (chr11:31823309 C > T, hg19)), which cosegregated with the phenotype of this pedigree. All the three patients (a pair of fraternal twins and their mother) exhibited bilateral FH and anterior segment dysgenesis (ASD) including microcornea, sclerocornea, obvious symmetrical corectopia, iris stromal dysplasia, goniodysgenesis, and abnormal distribution of fundus blood vessels. The girl of the fraternal twins also demonstrated bilateral temporal deviation of lenses and abnormal tissue membrane connecting anterior chamber angle and lens anterior capsule in the right eye. The mother additionally showed apparent cataract bilaterally and cupping of the optic disc in her left eye.

**Conclusion:**

A novel missense variant in *PAX6* gene was detected in a Chinese pedigree demonstrating bilateral FH and ASD. It is really distinctive that the ASD involves almost all parts of the anterior segment, and bilateral symmetrical corectopia is the most perceptible sign. This study expands the phenotypic and genotypic spectrum of *PAX6*-associated ocular diseases, and facilitates the understanding of the crucial role that *PAX6* plays in the development of the eye. Meanwhile, *PAX6* could be considered as a candidate pathogenic gene of bilateral symmetrical corectopia.

## Background

The *PAX6* gene is essential for ocular development. Mutations of it can lead to a broad phenotypic spectrum including aniridia [1] and many non-aniridia phenotypes such as isolated foveal hypoplasia (FH), microphthalmia, anophthalmia and coloboma, Gillespie syndrome, and Peters anomaly [2]. FH is described as an anatomical absence of the foveal pit [3] with continuity of all neurosensory retinal layers in the presumed foveal area, [4] and usually related to reduced vision and infantile nystagmus [5–7]. FH is caused by disruption of the normal development of the fovea from fetal week 22–25 to 15–45 months after birth; [5, 8] it could often be divided into FH1 (autosomal-dominant) and FH2 (autosomal-recessive), and *PAX6* gene and *SLC38A8* gene were reported to be responsible for them respectively [9].

*PAX6*-associated FH could be accompanied with mild iridal abnormalities, [10, 11] however, there were few reports focusing on *PAX6*-associated FH and obvious symmetrical corectopia. Corectopia describes abnormalities of pupil position and shape, [12, 13] which could be both congenital and acquired [12, 14]. It could be observed in Axenfeld-Reiger anomaly, ectopia lentis et pupillae (ELP), sectorial iris atrophy or other colobomatous lesions [13, 15].

Here we report a Chinese pedigree in which a pair of fraternal twins (a boy and a girl) and their mother consulted for obvious bilateral symmetrical corectopia. Multiple abnormalities of the anterior segments and FH was identified. Whole exome sequencing (WES) found a novel missense variant in *PAX6* gene which cosegregated with the phenotype in this pedigree.

## Methods

A pair of fraternal twins and their mother visited the consulting room in Beijing Tongren Hospital in August 2021, complaining of bilateral symmetrical corectopia and low vision. Thus, comprehensive ophthalmological and physical examinations were performed for them, and gene detection was conducted in this pedigree since the symptoms exhibited obvious genetic tendency. This study adhered to the Helsinki Declaration and its later amendments. Written informed consents were obtained from all the participants or their guardians after exhaustive explanation of the study. The whole protocol was approved by the ethical review committee of Beijing Tongren Hospital, Capital Medical University.

### Clinical evaluation

The birth history and development of the twins were carefully asked and recorded. So did the family history and history of gestation and delivery. The anterior segments of the patients were inspected by slit-lamp biomicroscopy. Other routine examinations included visual acuity, intraocular pressure, and ocular motility. The adults underwent ophthalmic ultrasound, ultrasonic biomicroscopy (UBM), optical coherence tomography (OCT) for the posterior segments, and wide-field fundus imaging. For the pre-school fraternal twins, OCT for the anterior segments was performed instead of the UBM due to lack of coorperation. Gonioscopy and visual field test were also performed on the mother. Since the ophthalmic signs demonstrated obvious genetic tendency, the paternal grandmother, the maternal grandfather and his mother and four siblings were also invited to undergo comprehensive examinations of their eyes in local hospitals.

FH was distinguished into four grades according to morphology by OCT: grade 1 with shallow foveal pit, presence of outer nuclear layer (ONL) widening, presence of outer segment (OS) lengthening; grade 2 as grade 1 but absence of foveal pit; grade 3 as grade 2 but absence of OS lengthening; grade 4 as grade 3 but absence of ONL widening [6].

### Whole exome sequencing and bioinformatics analysis

**Fig. 1:**
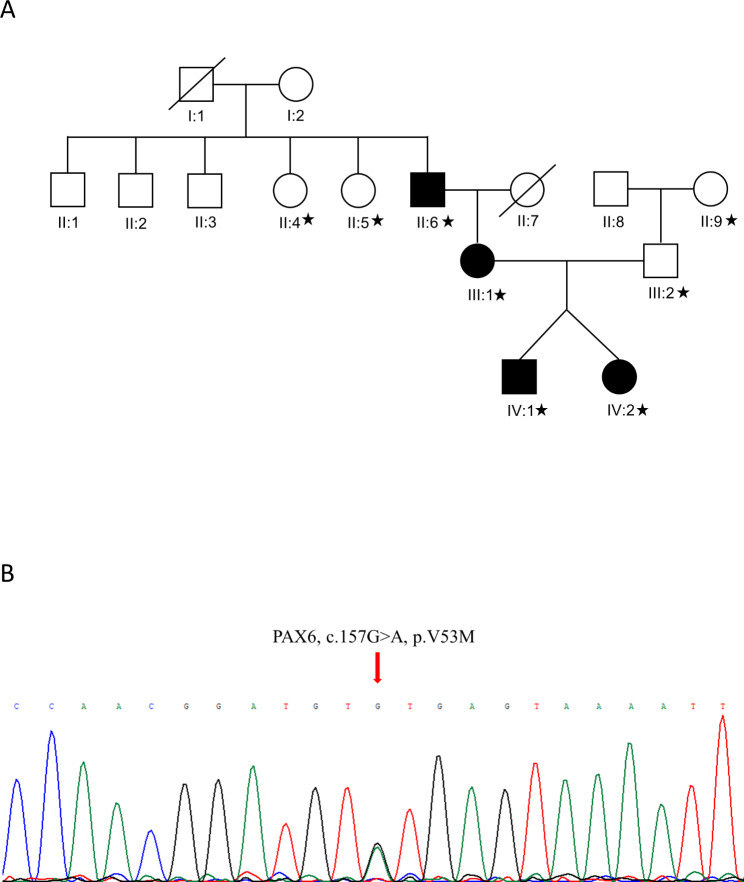
**A.** The pedigree of the family The fraternal twins, their mother, and their maternal grandfather were sufferers. Participants with asterisks provided peripheral blood samples for whole exome sequencing. **B.** *PAX6* c. 157G > A heterozygous mutation cosegregated with the clinical phenotype in this family

DNA was extracted from peripheral blood samples with TianGen Biochemical DNA isolation kit (Beijing Tiangen Biochemical Co., Ltd, Beijing, China) in eight members from the pedigree (Fig. 1A). The whole exome sequencing was performed at Beijing Novogene Bioinformatics Technology Co., Ltd (Beijing, China). Agilent 2100 was used to detect the fragment size of exon library, and 350–500 bp was considered to meet the requirements of computer. Illumina HiSeq 2500 was used for high-throughput sequencing, and the average sequencing depth was no less than 200X. Burrows Wheeler Aligner software was used to map the valid sequencing data to the human reference genome (hg19). The single nucleotide polymorphism and the insertions or deletions (Indels) were identified by the Samtools and bcftools separately. ANNOVAR program was performed to annotate the amino acid alternations. Variants were annotated using a variety of variant databases, such as dbSNP, Genome Aggregation Database (gnomAD), 1000 Genomes Project dataset, and the NHLBI-ESP project, and were filtered if MAF (minor allele frequency) > 1%. Nonsynonymous variants were evaluated according to scores of SIFT, Polyphen2, MutationTaster, CADD and FATHMM algorithms. ACMG guidelines were used to evaluate variants. Sanger sequencing was used for validation after the candidate pathogenic variants were found. Finally, species conservation of the mutant site was analyzed in several common animals with the UGENE software [16]. The PyMOL 2.0 Molecular System (Schrödinger, LLC, New York City, USA) was employed to determine functional changes in proteins in response to variants. And several online servers (I-Mutant 2.0, mCSM, SDM, DUET, DynaMut, PopMuSic and MAESTRO), based on Gibbs free energy (indicated by a ΔΔG value), were used to assess the stability of each variant.

## Results

The twins were born with cesarean at 36 weeks of gestational age. Birth weight was 2300 g for the girl and 2650 g for the boy. No special events were recorded in the history of gestation and delivery. No prominent abnormalities were found through comprehensive physical examinations in all the three patients.

### Ophthalmic characteristics

#### The girl of the fraternal twins

**Fig. 2:**
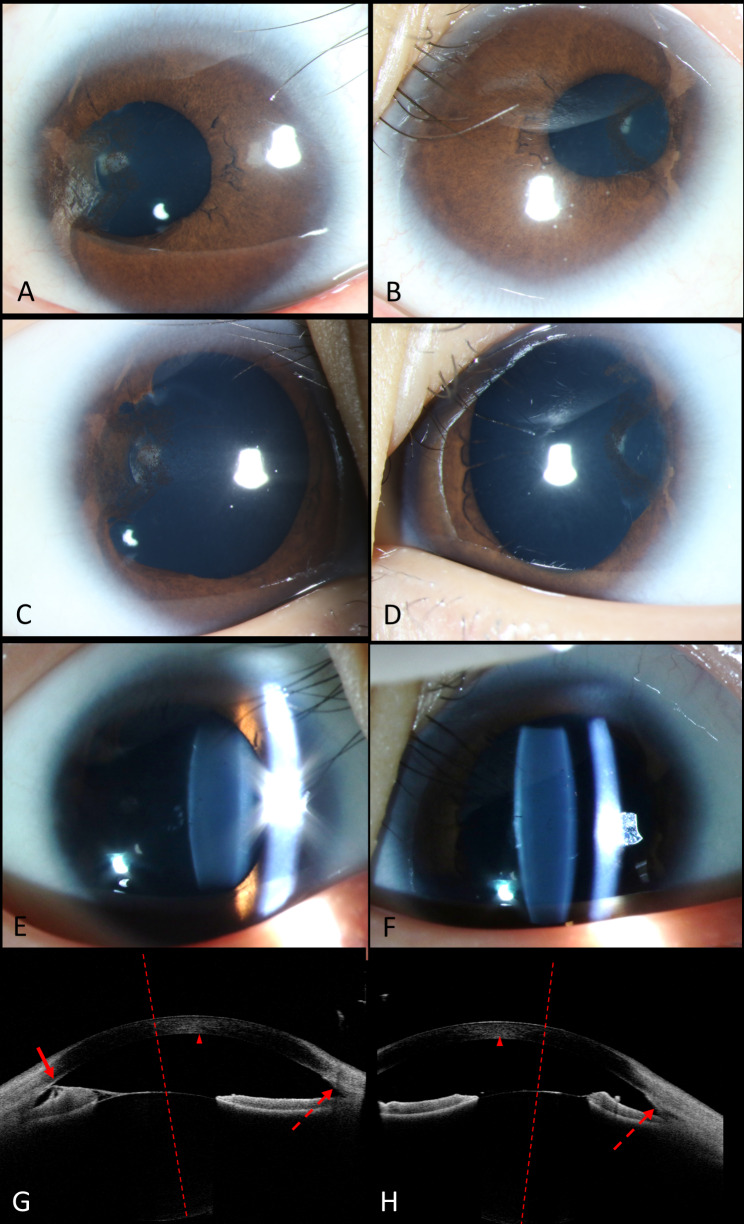
Anterior segments of the twin-girl. (Left column for the right eye and right column for the left eye.) **A-B**. Bilateral microcornea (7*8mm) and sclerocornea was presented. Both of the pupils were irregular-shaped and symmetrically displaced superiorly and temporally. **C-D**. Both pupils were poorly dilated and threadlike pigmentary persistent membrane was presented in the left eye. E-F. Both lenses presented mild patchy opacity in the posterior capsule. **G-H**. The anterior optical coherence tomography image of both eyes. Both pupils were temporally displaced and abnormal pigmentary tissue membrane could be observed on the temporal side connecting anterior chamber angle and lens anterior capsule in the right eye (arrow). Abnormal tissue membrane climbs from the root of iris to the corneal endothelium of anterior chamber angle (dotted arrow). The dotted line, which was drawn vertical to the tangent line of the posterior surface of the lens, was temporal to the center of the cornea (arrow head), showing that the lens was temporally displaced

**Fig. 3:**
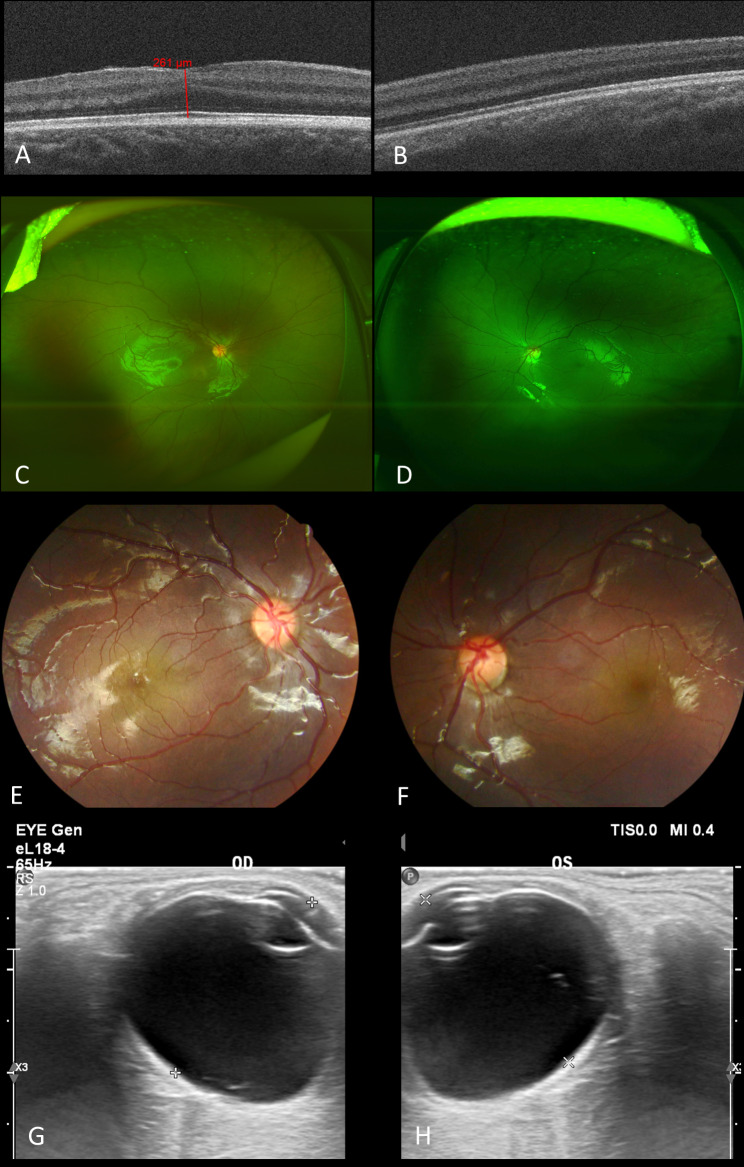
Posterior segments of the twin-girl. (Left column for the right eye and right column for the left eye.) **A-B**. Optical coherence tomography of the macula. The right eye exhibited grade 1 FH with a shallow foveal pit measuring 261 μm of retinal thickness, presence of ONL widening, and presence of OS lengthening, and the left eye demonstrated grade 4 FH with absence of foveal pit, ONL widening, and OS lengthening. **C-F**. The optic discs demonstrated normal size and colour, and the trunk of nasal retinal vein divided into upper and lower branches only after crossing the edge of optic disc. **G-H**. The axial length was 21.3 mm (right eye) and 21.1 mm (left eye) with no space-occupying lesions, vitreous opacity or retinal detachment

Both of her eye suffered mild myopia and the best-corrected visual acuity (BCVA) was 0.2 (right eye) and 0.15 (left eye). She demonstrated normal intraocular pressure and eye position. Horizontal nystagmus was obviously observed in both of her eyes. The slit-lamp biomicroscopy showed bilateral microcornea with diameters of 7 and 8 mm in the horizontal and vertical meridians respectively, sclerocornea especially on the temporal side, and a smooth surface of iris without crypts and contraction furrows (Fig. 2A-F). Both of her pupils were irregular-shaped, symmetrically displaced superiorly and temporally (Fig. 2A, B), dully reacted to light and poorly dilated (Fig. 2C-F). Abnormal pigmentary tissue membrane could be observed on the temporal side connecting anterior chamber angle and lens anterior capsule (Fig. 2C, D). Both lenses presented mild patchy opacity in the posterior capsule (Fig. 2E, F). The optic discs demonstrated normal size and colour, while the trunk of nasal retinal vein divided into upper and lower branches only after crossing the edge of optic disc (Fig. 3C-F). Anterior OCT showed displacement of the pupil, abnormal tissue membrane climbing from the root of iris to the corneal endothelium of anterior chamber angle, and temporal displacement of the lens (Fig. 2G, H); posterior OCT demonstrated grade 1 FH with shallow foveal pit, presence of ONL widening, and presence of OS lengthening in her right eye (Fig. 3A), and grade 4 FH with absence of foveal pit, ONL widening, and OS lengthening in the left eye (Fig. 3B). The axial length was 21.3 mm (right eye) and 21.1 mm (left eye) (Fig. 3G, H) .

### The boy of the fraternal twins

**Fig. 4:**
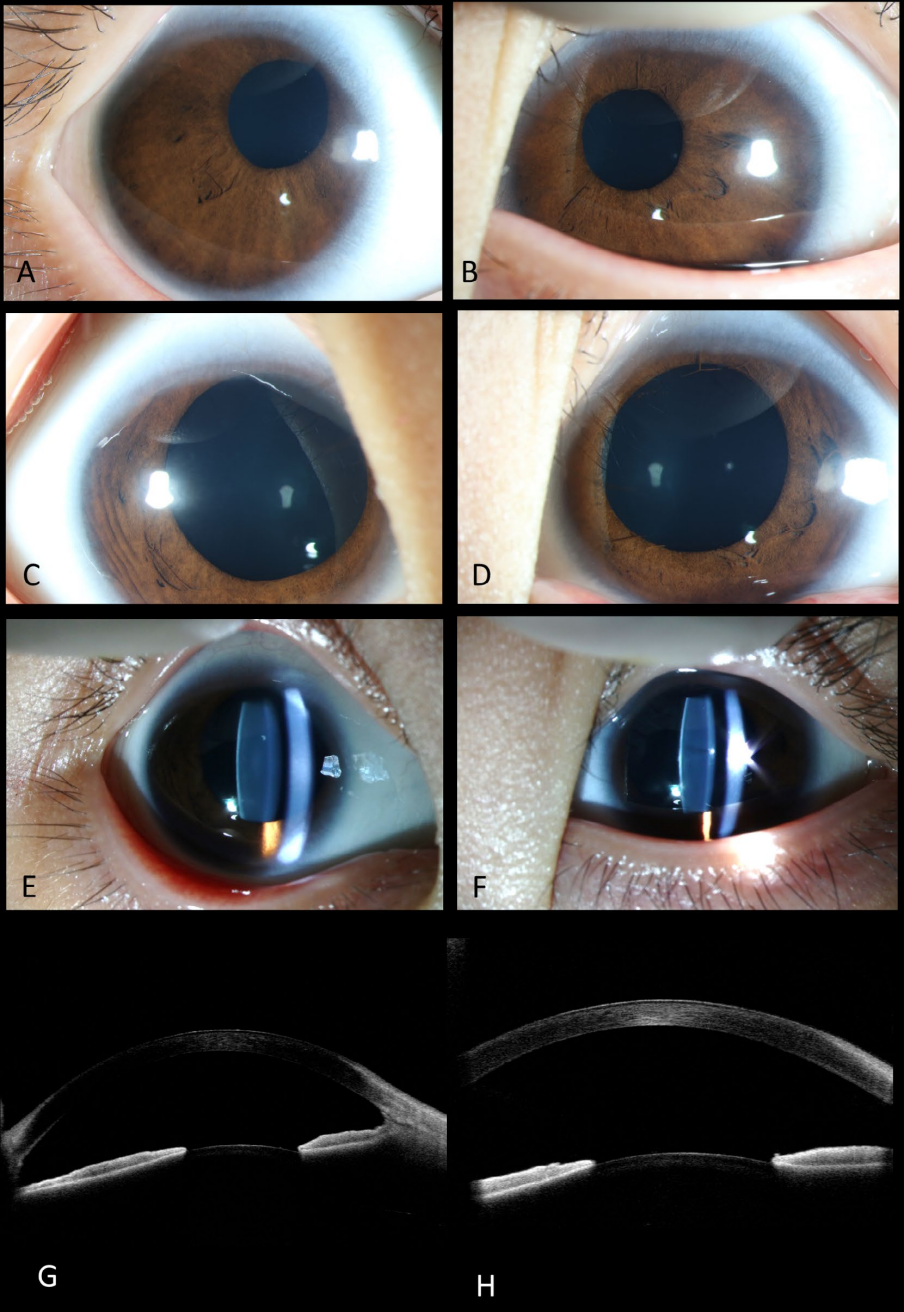
Anterior segments of the twin-boy. (Left column for the right eye and right column for the left eye.) **A-B**. Corneal diameters were 10 mm and 9 mm in the horizontal and vertical meridians, respectively, and sclerocornea was presented. Both of the pupils were almost oval but displaced superiorly and nasally. **C-D**. Both pupils were relatively poorly dilated and threadlike persistent membrane was presented only at 12 o’clock in the left eye. **E-F**. The reflection of the posterior capsule of the left lens was higher than normal with side light by slit-lamp biomicroscopy. **G-H**. Anterior OCT showed pupillary displacement and abnormal tissue membrane climbing from the root of iris to the corneal endothelium of anterior chamber angle

**Fig. 5:**
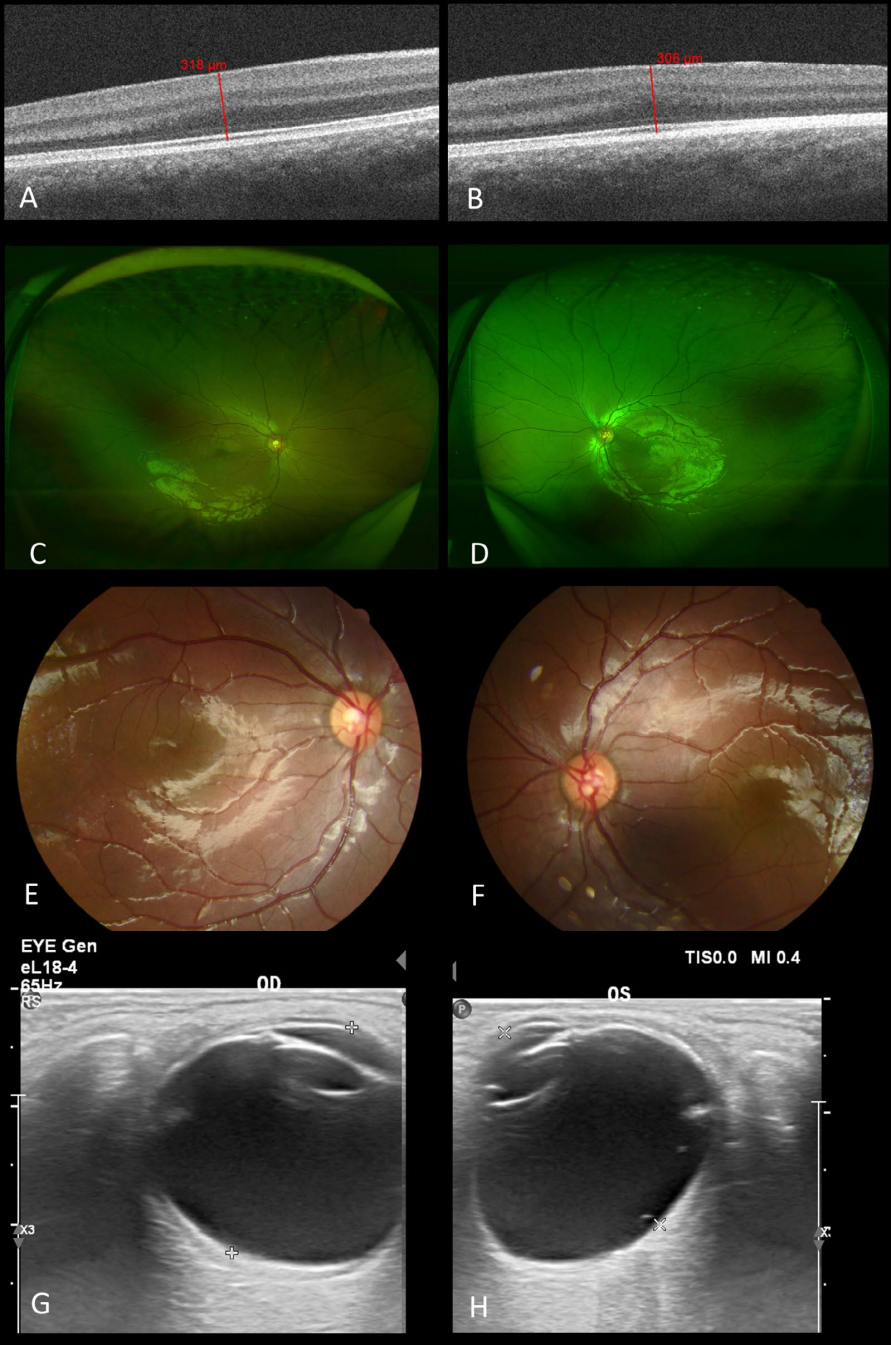
Posterior segments of the twin-boy. (Left column for the right eye and right column for the left eye.) **A-B**. Optical coherence tomography of the macula. Both of his fovea exhibited grade 3 FH with only ONL widening, foveal retinal thickness measuring 318 μm and 306 μm respectively. **C-F**. The optic discs demonstrated normal size and colour, and the trunk of nasal retinal vein divided into upper and lower branches only after crossing the edge of optic disc in both eyes. **G-H**. The axial length was 21.7 mm (right eye) and 21.6 mm (left eye) with no space-occupying lesions, vitreous opacity or retinal detachment

The even-aged twin-boy exhibited better vision than his sister, though lower than the normal value, with BCVA of 0.7 (right eye) and 0.3 (left eye). He demonstrated mild hyperopia, 10 radians of esotropia and normal intraocular pressure. Mild horizontal nystagmus was observed. Corneal diameters were 10 mm and 9 mm in the horizontal and vertical meridians respectively, and the sclerocornea was relatively more obvious on the nasal side (Fig. 4A, B). The iris exhibited a smooth surface with fractures (Fig. 4A, B). His pupils were almost oval. They symmetrically displaced superiorly and nasally, acted relatively dully to light, and dilated relatively poorly but better than those of his sisters (Fig. 4C, D). Threadlike persistent membrane could be observed in the left pupil (Fig. 4D). The reflection of the posterior capsule of the left lens was higher than normal with side light by slit-lamp biomicroscopy (Fig. 4E, F). Anterior OCT showed bilateral pupillary displacement and similar tissue membrane to his sister (Fig. 4G, H). Abnormal retinal vein routing also presented (Fig. 5C-F). Both of his fovea exhibited grade 3 FH with only ONL widening (Fig. 5A, B). Axial length was 21.7 mm (right eye) and 21.6 mm (left eye) (Fig. 5G, H) .

### The mother of the twins

**Fig. 6:**
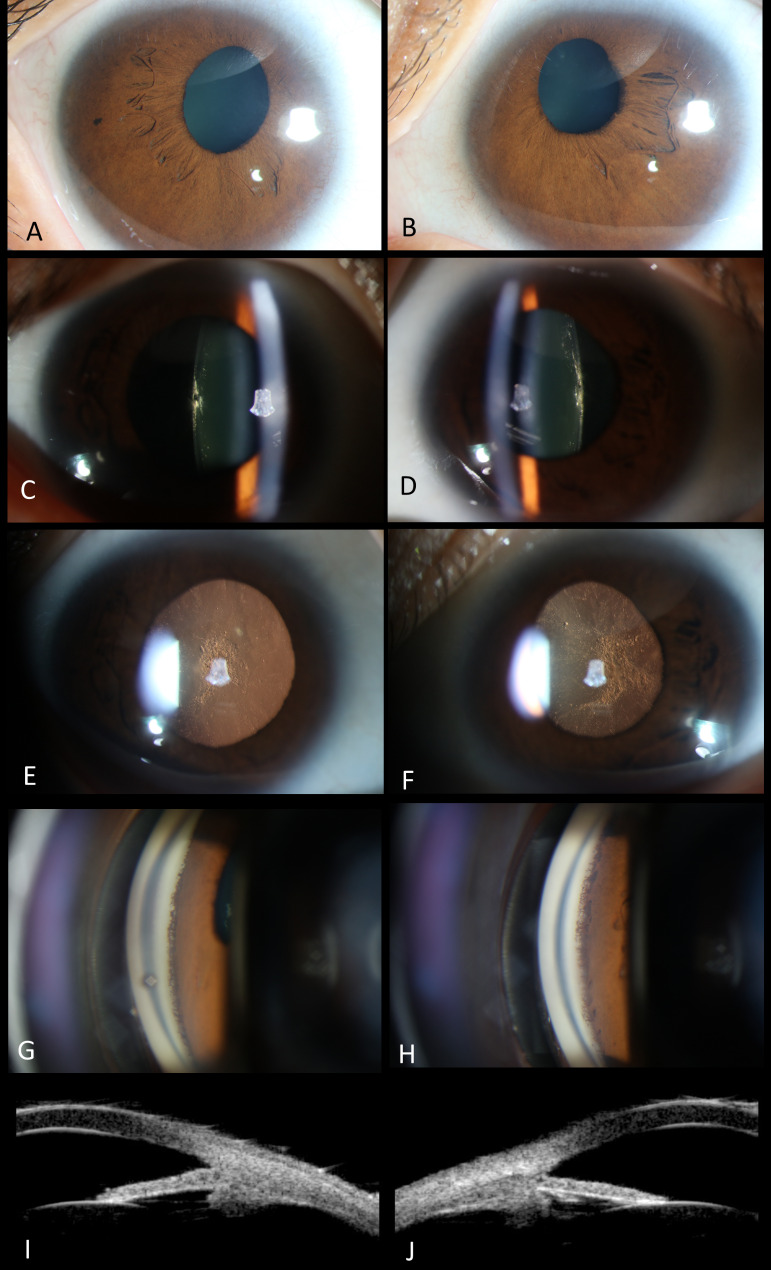
Anterior segments of the mother. (Left column for the right eye and right column for the left eye.) **A-B**. Bilateral microcornea (10*10mm) and corneal scleralization was presented. Both of the pupils were irregular-shaped and displaced superiorly and nasally. Texture of the temporal iris was sparse in both of her eyes. **C-F**. Bilateral opacity could be observed in the central posterior capsule and the cortex beneath it. **G-H**. Dense comb-like iris roots stretching beyond the scleral spurs and inserting anterior to the Schwalbe line could be observed by gonioscopy in both eyes. Only mild pigment was revealed. **I-J**. UBM showed open anterior chamber angle, vague definition of the ciliary processes, and normal location of the lens. The scleral spurs were covered with high-echo tissue, which was consistent with the dense comb-like iris roots revealed by gonioscopy

**Fig. 7:**
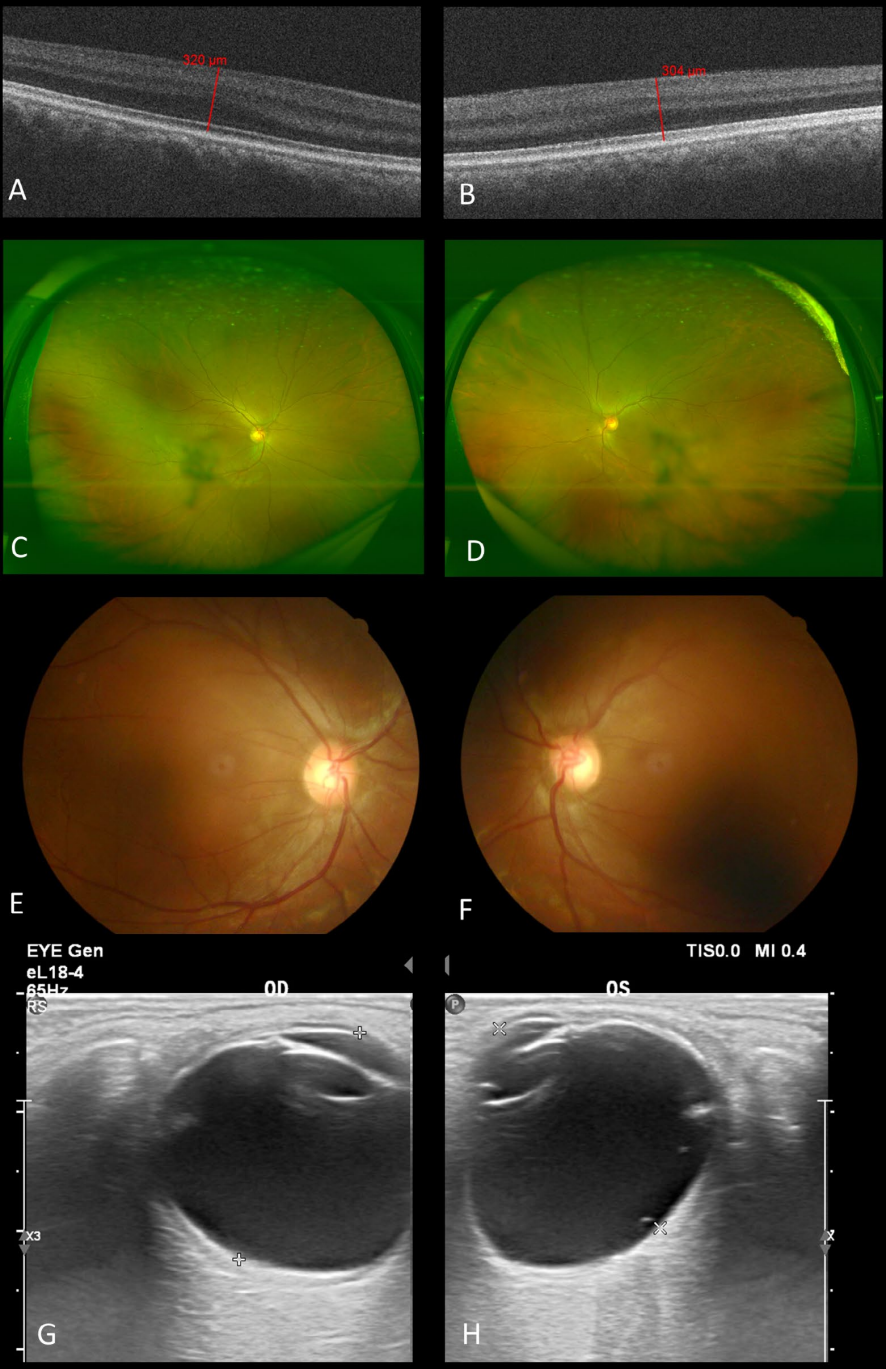
Posterior segments of the mother. (Left column for the right eye and right column for the left eye.) **A-B**. Optical coherence tomography of the macula. Both eyes exhibited grade 3 FH with only ONL widening, foveal retinal thickness measuring 320 μm and 304 μm respectively. **C-F**. Bilateral opacity of the vitreous and cupping of the left optic nerve head (cup/disc ratio about 0.5) was presented. The trunk of nasal retinal vein divided into upper and lower branches only after crossing the edge of optic disc. **G-H**. Ophthalmic ultrasound showed bilateral vitreous opacity and the axial length was 21.9 mm (right eye) and 21.8 mm (left eye). No space-occupying lesions or retinal detachment was observed

She was 32 years old with low vision (BCVA: 0.2, both eyes), also demonstrated mild myopia, esotropia (+ 15°), horizontal nystagmus, and normal intraocular pressure. The cornea measured 10 mm in both horizontal and vertical meridians, and sclerocornea could also be observed. Her pupils were irregular-shaped and symmetrically displaced superiorly and nasally (Fig. 6A, B). Both of her pupils reacted dully to light and were poorly dilated, but there was no persistent membrane observed (Fig. 6C, D). No crypts or contraction furrows were observed in her smooth iris and texture of the temporal side was sparse with fracture (Fig. 6A, B). For both of her lenses, opacity could be observed in the central posterior capsule and the cortex beneath it(Fig. 6C-F). Gonioscopy revealed mild pigment but dense comb-like iris roots stretched beyond the scleral spurs and inserted anteriorly to the Schwalbe line (Fig. 6G, H). UBM showed open anterior chamber angle, vague definition of the ciliary processes, and normal location of the lens. The scleral spurs were covered with high-echo tissue (Fig. 6I, J). Ophthalmic ultrasound showed bilateral vitreous opacity and the axial length was 21.9 mm (right eye) and 21.8 mm (left eye) (Fig. 7G, H). Fundus imaging revealed no prominent abnormalities except cupping of the left optic nerve head (cup/disc ratio about 0.5) and similar abnormal retinal vein routing to her children (Fig. 7C-F). Humphry visual field test exhibited corresponding general reduction of sensitivity in her left eye. Posterior OCT also exhibited grade 3 FH bilaterally (Fig. 7A, B).

The clinical characteristics of the three patients above are listed in Table 1. Figure 1 A demonstrates the pedigree of the family. The maternal grandfather of the fraternal twins suffered from microcornea and low vision since his childhood, underwent surgical treatment for cataract in his early thirties, and maintain visual acuity around 0.2. No prominent ocular abnormalities were found in the eyes of all his five siblings after comprehensive ophthalmological examinations in local hospitals. Comparing with the boy, the girl and their mother exhibited additional lens abnormity besides FH, with the girl demonstrating mild opacity of the cortex and dislocation of the lenses, and the mother presenting opacity of the cortex and the posterior capsule of the lenses.

**Table 1:** Baseline Characteristics of The Family

			Twin-girl	Twin-boy	Mother
Age (years)			5	5	32
Sex			Female	Male	Female
Physical Examination			-	-	-
BCVA (OD/OS)			0.2/0.15	0.7/0.3	0.2/0.2
Refractive Diopter (OD/OS)			-0.50Ds + 1.75Dc×95/ -1.50Ds + 3.50Dc×95	+ 2.50Ds + 2.25Dc×80/ +0.75Ds + 2.75Dc×110	-2.25Ds + 2.00Dc×80/ -2.25Ds + 2.00Dc×100
IOP (OD/OS, mmHg)			16/17	15/15	13/16
Eye Position			Normotopia	Esotropia	Esotropia
Nystagmus			+	+	+
Cornea			Microcornea; limbal scleralization	Microcornea; limbal scleralization	Microcornea; limbal scleralization
Pupil	Shape		Irregular	Oval	Irregular
	Displacement		Superiorly and temporally	Superiorly and nasally	Superiorly and nasally
	Persistent Membrane		+	+	-
	React to Light		Dully	Dully	Dully
	Dilated		Poorly	Poorly	Poorly
Anterior Chamber Angle			Abnormal tissue membrane	Abnormal tissue membrane	Anterior insertion of iris roots
Iris			Smooth surface	Smooth surface	Sparse texture (temporal)
Lens	Opacity		Posterior capsule	Posterior capsule (OS)	Posterior capsule and cortex
	Displacement		Temporally	-	-
Posterior Segments		Fovea	Dysplasia grade1(OD) grade4(OS)	Dysplasia grade3 (OU)	Dysplasia grade3 (OU)
		Else	-	-	Cupping of the optic-nerve head (OS) and vitreous opacity (OU)

* BCVA, best-corrected visual acuity; IOP, intraocular pressure

#### Variant detection

**Fig. 8:**
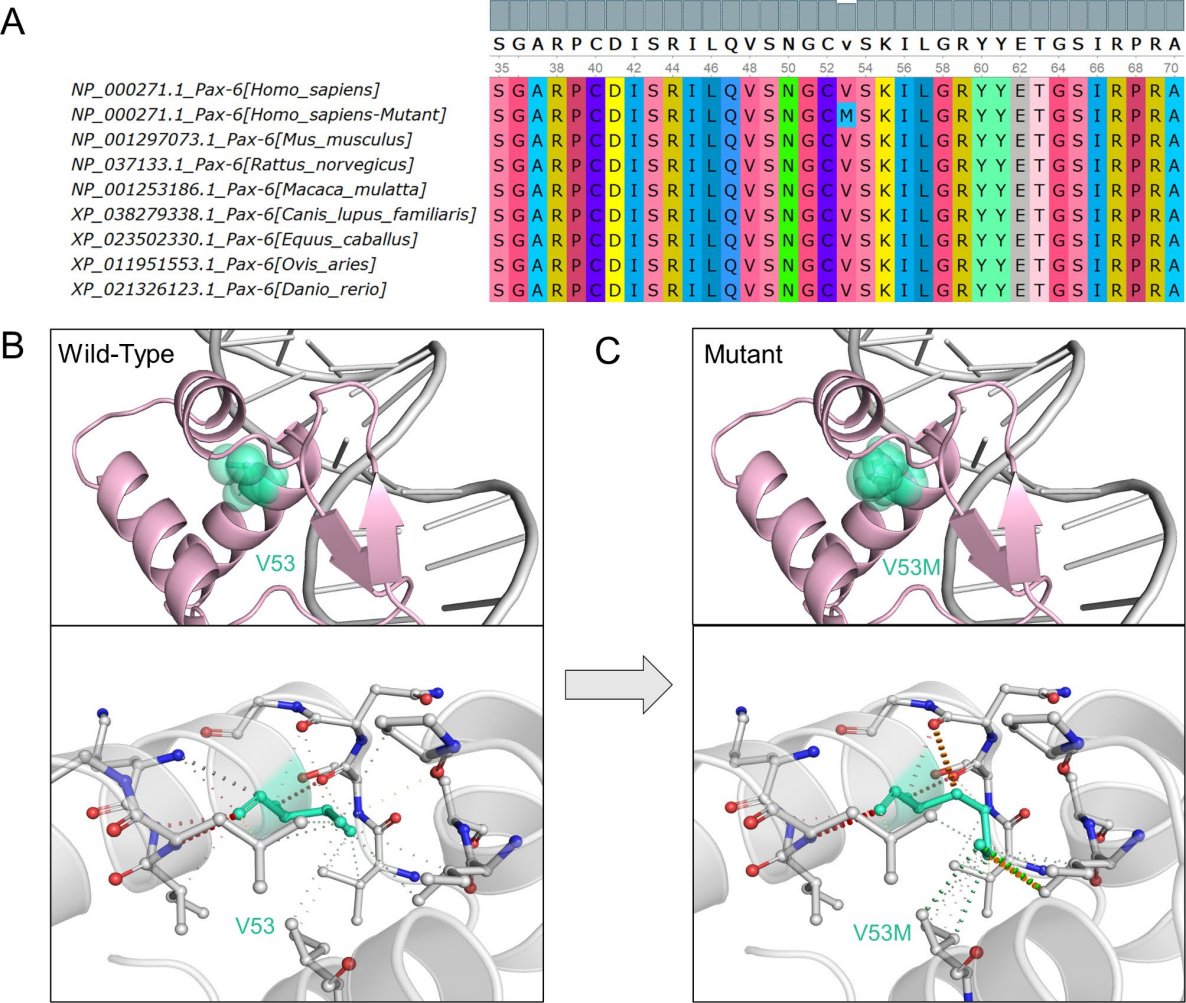
Analysis of the evolutionary conservation and the protein structural stability of *PAX6*. (**A**) The *PAX6* protein alignment among 8 different species demonstrates the evolutionary conservation of residue V53. (**B-C**) 3D protein structure of human *PAX6* (predicted by AlphaFold) shows the conformational changes induced with and without the p.(Val53Met) substitution

WES and bioinformatic analysis revealed a novel heterozygous missense variant in *PAX6* (NM_000280.5:c.157G > A, p.(Val53Met) (chr11:31823309 C > T, hg19)). This variant cosegregated with the phenotype in this pedigree (Fig. 1B), and was validated by Sanger sequencing. According to the ACMG genetic variant classification criteria and guidelines, the *PAX6* gene missense variant p.(Val53Met) met 2 PM evidence and 2 PP evidence, which confirmed that this variant was likely pathogenic. In this variant, valine was substituted by methionine at position 53 in *PAX6* (p.(Val53Met)), which was not yet present in the gnomAD, 1000 Genomes Project dataset, or the NHLBI-ESP project. A variety of bioinformatics software predicted that the p.(Val53Met) substitution had a deleterious or disease-causing effect. In addition, evolutionary conservation analysis by phyloP showed that p.(Val53Met) substitution resulted in highly conserved amino acid changes, which was also analyzed in several common animals through the UGENE software (Fig. 8A). Protein-function prediction suggested that the V53 position was substituted by the methionine with a longer side chain, resulting in the crowded conformation and changes of hydrophobic contacts with other residues (Fig. 8B-C). This may alter the spatial conformation of the helical domain that interacts with the DNA, thereby affecting the binding of the protein and the DNA. For computational-stability analysis, the 3D protein structure of human *PAX6* (predicted by AlphaFold) was used to assess the stability of the p.(Val53Met) variant. Results indicated that this substitution induced notable destabilization with a large decline in Gibbs free energy determined by five different types of online servers (Table 2).

**Table 2:** The Stability Analysis of Mutations by Different Web Servers

Mutation		I-Mutant 2.0	mCSM	SDM	DUET	ENCoM
*PAX6*-V53M	ΔΔG(kcal/mol)	-0.94	-1.083	-0.630	-1.058	0.224
	Prediction	Destabilizing	Destabilizing	Destabilizing	Destabilizing	Destabilizing

## Discussion

According to previous reports, the most common genetic etiology for typical FH was albinism-associated genes (67.5%), followed by *PAX6* (21.8%), *SLC38A8* (6.8%), and *FRMD7* (3.5%) variants [17]. In this study, we presented a Chinese pedigree demonstrating bilateral FH with a novel missense variant in *PAX6* (p.(Val53Met)), which we called *PAX6*-associated FH, accompanied by variable anterior segment dysgenesis (ASD) mainly characterized by symmetrical corectopia.

*PAX6* is most well-known as the causative gene of aniridia. It encodes a protein who has two DNA-binding domains, the paired domain (PD) and the homeodomain (HD) [1]. The PD consists of N-terminal (NTS) and C-terminal subdomains (CTS), that can bind the consensus DNA sequences and influence the DNA-binding effect of the HD [11, 18]. Part of the missense variants contribute to isolated FH and are mostly located in CTS, while those contribute to aniridia, microphthalmia, anophthalmia, and coloboma are mainly located in NTS [11, 19]. The missense variants could cause differences in DNA binding, protein folding, and the transactivation activities of *PAX6* [1, 11]. Therefore, as is shown in the Result section, we preliminarily believed that the p.(Val53Met) variant found in this study caused the phenotype by reducing the structural stability of PAX6 protein.

Previous reports showed that mutants of *PAX6* could cause similar manifestations to our cases. Simultaneous keratopathy, absence of iris, and cataract were reported to be associated with c.619 A > T, [20] c.112 C > G, [21] and c.214G > C in *PAX6* [21]. Mishra et al. had reported the PD mutants of *PAX6* (L46R, C52R, and V53L) in a family exhibiting aniridia and cataracts but without predominant abnormality in the posterior segments in 2002 [22]. A later review showed that isolated FH could also be associated with *PAX6* missense mutations in PD: c.227 C > G, and c.382 C > T.^1^ Matsushita I et al. also reported c.1095T > G mutation in a patient with FH and normal anterior segments and c.58 C > T mutation in patients with FH and posterior polar cataract [9]. However, cases of our report exhibited almost all the manifestations of the anterior segments besides FH, including microcornea, sclerocornea, obvious symmetrical corectopia, iris stromal dysplasia, and goniodysgenesis.

Actually, in most cases, *PAX6*-associated FH could be accompanied with variable iris changes: You B and colleagues found that 93% of the patients with aniridia and *PAX6* mutations presented FH in their cohort; [23] a missense variant was also identified by Hingorani et al. in patients with FH and mild structural abnormalities in the iris; [1] Jiang Y and colleagues reported that in their observation of FH, full iris with subtle structural abnormalities with mutations in the C-terminal region of the PD of *PAX6* [11]. In our study, none of the three patients demonstrated complete or partial absence of the iris, but abnormalities of the iris could still be observed. That is to say, phenotype of this variant could be associated with variable iris changes characterized by corectopia and iris stromal dysplasia instead of absence of iris. The relatively central position of pupil appears to be determined by equal traction centrifugally in all directions by the dilator muscle of the iris, [12] and pupil displacement occurs as a result of iris atrophy [15]. Thus, from another perspective, these are all different degrees and phenotypes of iris changes, which is consistent with the experience that missense variants of *PAX6* are usually associated with milder but atypical phenotypes [1].

Meanwhile, in addition to iris changes, all the three patients also demonstrated other anterior segment abnormalities including microcornea, sclerocornea and goniodysgenesis (including abnormal tissue membrane in the fraternal twins and dense comb-like iris roots inserting anterior to the Schwalbe line in the mother), and presenile cataract was observed in the lenses of the two females. That is to say, phenotype of this variant could demonstrate bilateral FH and ASD involving almost all parts of the anterior segment including cornea, iris, anterior chamber and lens, which distinguishes this variant from previously reported ones.

Apart from the variant (p.(Val53Met)) identified in this study, Grønskov K and colleagues reported a female with the variant p.(Val53Leu) demonstrated circumpupillary iris aplasia with fine strands crossing the pupillary area; [24] In the study of Cross E et al., a male with the variant p.(Val53Gly) exhibited cataracts and nystagmus [25]. Although these single nucleotide variants above affected the same amino-acid residue, they led to different phonotypic presentations. Further studies may detect a broader clinical spectrum.

Given the symmetrical dislocation of the pupils and lenses present in the twin-girl, we once take the diagnosis of ELP into consideration before gene detection. Typical ELP is characterized by symmetrically displaced pupils and lenses, in which the pupils are generally displaced in a direction opposite to that of the ectopic lenses [26, 27]. Recent years, diagnosis of ELP has been broadened to ectopic lenses, the imperative sign, with pupillary abnormalities such as ectopia pupillae, persistent pupillary membrane, iris transillumination, and poor pupillary dilation, [28, 29] and mutations of the gene *ADAMTSL4*, which was inherited autosomal-recessively was found responsible for this entity [30–32]. In our study, only the twin girl presented manifestations of the anterior segments consistent with ELP, the boy and their mother exhibited no ectopic lenses. More importantly, their autosomal-dominant inheritance pattern was contrary to that of ELP. Therefore, the diagnosis of ELP was ruled out after gene detection.

Presence of FH is usually the main reason for low vision because increase of the central foveal thickness reduces the maximum cone density [33]. The mother and the twin-girl in this study exhibited abnormalities of the pupils and lenses apart from the fovea, which are essential in the optical pathway, so it is not surprising that they suffered from low vision. However, visual acuity of patients with FH could range from 20/20 to 20/400, and was correlated with extent of the FH [4]. This could be explained since foveal cone specialization can be preserved both anatomically and functionally despite the absence of a pit, [4] and the maturation of the cone photoreceptors during the development of the fovea is an independent phenomenon [9]. Matsushita I et al. also suggested that eyes with absence of the foveal pit in the OCT images which was caused by *PAX6* mutations could be associated with fairly good visual acuity [9]. Given these, the relatively good vision of the right eye of the twin-boy may be reasonable.

All the three patients exhibited goniodysgenesis, and the mother has already demonstrated cupping of the optic nerve head in her left eye, which showed relatively denser comb-like iris processes attaching anterior to the Schwalbe line. Even general sensitivity of her left eye has been decreased as shown in the visual field test. Moreover, the mother has already demonstrated prominent cataract bilaterally in her early thirties, and the twin-girl also exhibited opacity of the cortex of the lens. Although the twin-boy showed no abnormality in his lenses, goniodysgenesis was also observed. Thus, sufferers of this mutation may be high-risk groups of secondary glaucoma and cataract; close ophthalmological monitoring since childhood is of necessity. Meanwhile, with high clinical heterogeneity, gene screening is recommended for diagnosis.

## Data Availability

The datasets used and analysed has been deposited in GenBank (BioProject ID PRJNA894466).

## List of Abbreviations

ASD: Anterior segment dysgenesis
BCVA: Best-corrected visual acuity
CTS: C-terminal subdomain
ELP: Ectopia lentis et pupillae
FH: Foveal hypoplasia
HD: Homeodomain
NTS: N-terminal subdomain
OCT: Optical coherence tomography
ONL: Outer nuclear layer
OS: Outer segment
PD: Paired domain
UBM: Ultrasonic biomicroscopy
